# A New Inner Heliosphere Proton Parameter Dataset from the *Helios* Mission

**DOI:** 10.1007/s11207-018-1377-3

**Published:** 2018-11-26

**Authors:** David Stansby, Chadi Salem, Lorenzo Matteini, Timothy Horbury

**Affiliations:** 1 Department of Physics, Imperial College London, London, SW7 2AZ UK; 2 Space Sciences Laboratory, University of California, Berkeley, CA 94720 USA; 3 LESIA, Observatoire de Paris, Université PSL, CNRS, Sorbonne Université, Univ. Paris Diderot, Sorbonne Paris Cité, 5 place Jules Janssen, 92195 Meudon, France

**Keywords:** Solar wind, Heliosphere, Inner heliosphere, Solar wind protons

## Abstract

In the near future, *Parker Solar Probe* and *Solar Orbiter* will provide the first comprehensive *in-situ* measurements of the solar wind in the inner heliosphere since the *Helios* mission in the 1970s. We describe a reprocessing of the original *Helios* ion distribution functions to provide reliable and reproducible data to characterise the proton core population of the solar wind in the inner heliosphere. A systematic fitting of bi-Maxwellian distribution functions was performed to the raw *Helios* ion distribution function data to extract the proton core number density, velocity, and temperatures parallel and perpendicular to the magnetic field. We present radial trends of these derived proton parameters, forming a benchmark to which new measurements in the inner heliosphere will be compared. The new dataset has been made openly available for other researchers to use, along with the source code used to generate it.

## Introduction

With the imminent launches of *Parker Solar Probe* (Fox *et al.*, 2016) and *Solar Orbiter* (Müller *et al.*, 2013), heliospheric and solar physics are about to enter a new age of discovery. To date, the most comprehensive mission to visit the inner heliosphere and make *in-situ* measurements of the solar wind was the *Helios* mission, consisting of two spacecraft, which explored the heliosphere from 0.29 – 1 AU in the 1970s and 1980s, covering solar minimum between Solar Cycles 20 and 21 and the maximum of Solar Cycle 21 (Porsche, 1977). The data returned by these spacecraft provided a wealth of information, but the computational resources available to process the data were limited at the time, and there is no publicly available dataset containing reliable and reproducible moments derived from the full three-dimensional (3D) distribution functions. In this paper we revisit the plasma measurements made on board the two *Helios* spacecraft. The plasma data have been reprocessed before (*e.g.* Marsch, Ao, and Tu, 2004; Matteini *et al.*, 2007; Hellinger *et al.*, 2011), but importantly, the new dataset and the code used to generate it are openly available to researchers. This makes the dataset easily reproducible and reusable.

The solar wind primarily consists of protons, with a smaller fraction of alpha particles ($\sim 1\%$ – 5% by positive ion number density), a series of other minor ions ($\ll 1\%$ by positive ion number density), and neutralising electrons (Neugebauer and Snyder, 1962; Marsch *et al.*, 1982a; Pilipp *et al.*, 1987; Bochsler, 2007). The proton population can be further split into two: the proton core, which accounts for $\approx 90\%$ of the protons, and the smaller proton beam, which travels at a different velocity to the core (Feldman *et al.*, 1973; Marsch *et al.*, 1982b). Here we present systematic bi-Maxwellian fits to the proton core population for the entire duration the *Helios* mission.

In Section 2 we give a brief overview of the data that were already widely available to researchers. We provide in Section 3.1 an overview of the plasma instrumentation, and in Section 3.2, we describe the data processing and summarise the new dataset. In Section 4 we compare the dataset to the previously available data. In Section 5 we use the new dataset to provide a new set of radial trends for the proton core population of the solar wind.

## Previously Available Data

As far as we know, the only other publicly available set of proton plasma parameters available from the *Helios* mission is the “merged” dataset.^1^ This set of parameters was calculated in the 1970s and 1980s by taking numerical moments of 1D energy spectra, obtained by integrating the 3D distributions over all solid angles. Although taking numerical moments is computationally fast, it has a number of differences when compared with a bi-Maxwellian analysis of the full 3D distribution function:

- Only the total number density is calculated. This does not distinguish between the proton core and beam populations. If the total proton number density is of interest, then a moment is accurate, but to obtain the difference between the number density of the core or beam, an analytical fitting process must be used.
- Only the component of the temperature tensor in the radial direction ($T _{\mathrm{r}}$) is calculated. For a bi-Maxwellian with two true temperatures ($T _{\perp }$ and $T_{\parallel }$)$,T_{\mathrm{r}}$ depends in a non-trivial way on both $T_{\perp }$ and $T_{\parallel }$, but also on the angle that the instantaneous magnetic field vector ($\mathbf{B}$) makes with the radial direction (Maruca, 2012, Section 2.2). This leads to correlations of $T_{\mathrm{r}}$ with $\mathbf{B}$ that are not real, but instead are built in to the definition of $T_{\mathrm{r}}$ as calculated from a moment of an energy spectrum (*e.g.* Gogoberidze, Voitenko, and Machabeli, 2018).

In the rest of this paper we use moment to refer to the previously available data, and corefit to refer to the reprocessed dataset described here. We note that to calculate the number density, our method of reprocessing is not intrinsically better than taking moments, but instead provides a different set of information. However, to calculate temperatures, our method provides additional information that is not available from a simple moment analysis of energy spectra. In Section 4 we quantitatively compare data obtained using the two different methods.

## Data Processing

### Raw Data

Both *Helios* 1 and 2 were equipped with an experiment for measuring the distribution function of positively charged particles in the solar wind, called the E1 plasma instrument (Schwenn, Rosenbauer, and Miggenrieder, 1975). For much more detailed information, we refer to the instrument technical paper (Rosenbauer *et al.*, 1981).

The E1 experiment was an electrostatic analyser that counted particles as a function of their energy per charge ($E/q$). There were 32 $E/q$ channels logarithmically spaced between 0.155 keV/q and 15.3 keV/q, and nine angular elevation channels oriented perpendicular to the spin plane of the spacecraft (the ecliptic plane) and separated by 5^∘^. Resolution in the azimuthal direction was built up using the spin of the spacecraft, with measurements taken every 5^∘^. During each spin period, lasting 1 second, the flux in each angular bin was measured at a fixed $E/q$. Over 32 spins of the spacecraft, this allowed all 32 $E/q$ channels to be sampled in each angular direction. In high-resolution mode, a $7 \times 7$ grid of angular measurements in all 32 $E/q$ channels was transmitted back to Earth, centred around the distribution peak; in low-resolution mode, this was reduced to a $5 \times 5$ angular grid across nine $E/q$ channels, again centred on the distribution peak. Distributions transmitted in both modes contain enough data for locating and fitting to the proton core.

Because the E1 instrument had no mass discrimination, the 3D distribution functions contain contributions from both protons and alpha particles (Marsch *et al.*, 1982a). Because the protons and alphas are well separated in energy, and the protons form the majority of the distribution, it was simple to fit a bi-Maxwellian distribution to the protons alone.

Both spacecraft also had two magnetometers: the E2 experiment with data available at four vectors/second (Musmann *et al.*, 1975), and the E3 experiment with data available at one vector every 6 seconds (Scearce *et al.*, 1975). Magnetic field data were used as part of the fitting process to constrain the symmetry axis of the fitted bi-Maxwellian. For times when the higher rate E2 data were available, they were used, but otherwise, data from the E3 experiment were used.

### Fitting Process

**Figure 1:**
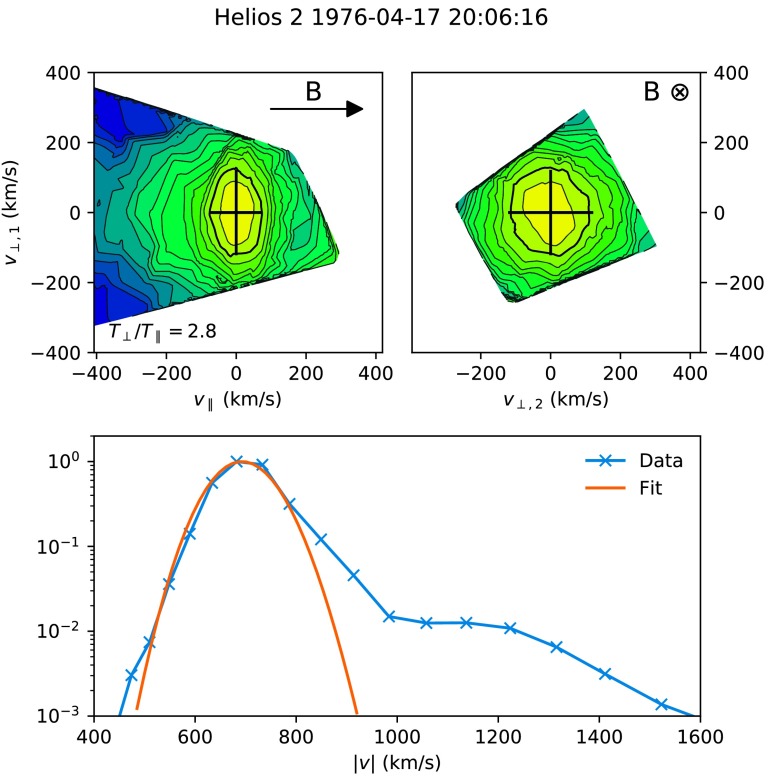
Example of a fast solar wind distribution function data and corresponding fit on 17 April 1976. The *top-left panel* shows a cut of the distribution function in a plane containing the local magnetic field ($\mathbf{B}$), centred at the bulk velocity. The *top-right panel* shows a cut in the plane perpendicular to $\mathbf{B}$, also centred at the bulk velocity. In both panels, contours are spaced logarithmically and the fitted thermal speeds in each direction are shown with *black crosses*. The $1/e$ contour is highlighted as a *thick black line*, which is located one thermal width away from the centre for a bi-Maxwellian. The *bottom panel* shows the experimentally measured distribution function (*blue line*) and fit (*orange line*) integrated over all solid angles, and normalised to the distribution function peak.

**Figure 2:**
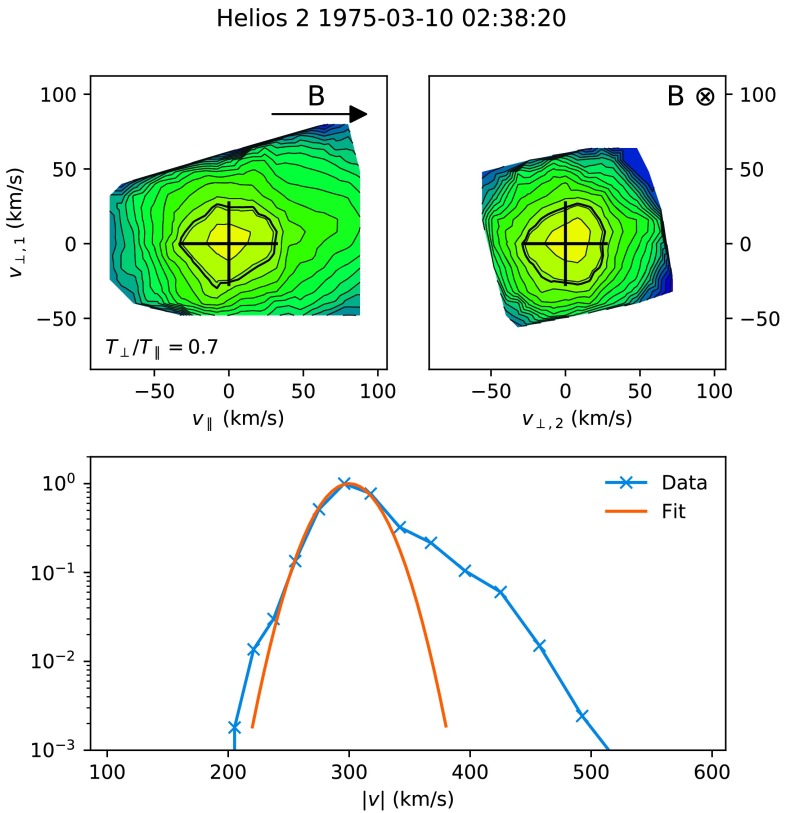
Example of a slow solar wind distribution function data and corresponding fit on 10 March 1975. The *top-left panel* shows a cut of the distribution function in a plane containing the local magnetic field ($\mathbf{B}$), centred at the bulk velocity. The *top-right panel* shows a cut in the plane perpendicular to $\mathbf{B}$, also centred at the bulk velocity. In both panels, contours are spaced logarithmically and the fitted thermal speeds in each direction are shown with *black crosses*. The $1/e$ contour is highlighted as a *thick black line*, which is located one thermal width away from the centre for a bi-Maxwellian. The *bottom panel* shows the experimentally measured distribution function (*blue line*) and fit (*orange line*) integrated over all solid angles, and normalised to the distribution function peak.
iii) The number density was calculated from
  3 $$ n = \underline{A} \cdot \pi ^{3/2}\underline{w}_{\perp }\underline{w} _{\perp }\underline{w}_{\parallel } , $$
  the two temperatures from
  4 $$ T_{\perp / \parallel } = \frac{m_{p}\underline{w}_{\perp / \parallel }^{2}}{2k_{B}} , $$
  and the bulk velocity fitted in the field aligned frame was rotated back to the heliocentric radial–tangential–normal (RTN) instrument frame of reference to give $(v_{\mathrm{r}}, v_{\mathrm{t}}, v_{\mathrm{n}})$.

Each experimentally measured distribution function was fitted with a bi-Maxwellian distribution function using the following process:

i) If magnetic field data were available from one of the magnetic field instruments, an average magnetic field ($\mathbf{B}$) was calculated from individual measurements that fell between the time of the first and last non-zero measurements in each individual distribution function. The distribution function was then rotated into a frame aligned with $\mathbf{B}$. This gave the rotated distribution function $f_{\mathrm{data}} \left ( v_{\parallel }, v_{\perp 1}, v_{\perp 2} \right )$, where $v_{\parallel }$ is the direction parallel to $\mathbf{B}$ and $v_{\perp 1,2}$ are two orthogonal directions to $\mathbf{B}$ in velocity space.
ii) The following 3D bi-Maxwellian function was fitted to the data (fit parameters underlined):
  1 $$ f_{\mathrm{fit}} ( v_{\parallel }, v_{\perp 1}, v_{\perp 2} ) = \underline{A} \cdot \exp - \biggl\{ \biggl( \frac{v_{\parallel } - \underline{u}_{\parallel }}{\underline{w}_{\parallel }} \biggr)^{2} + \biggl( \frac{v_{\perp 1} - \underline{u}_{\perp 1}}{\underline{w}_{\perp }} \biggr)^{2} + \biggl( \frac{v_{\perp 2} - \underline{u}_{\perp 2}}{\underline{w}_{\perp }} \biggr)^{2} \biggr\} . $$
  The six fit parameters were amplitude ($\underline{A}$), three bulk velocity components ($\underline{u}_{\parallel }$, $\underline{u}_{ \perp 1}$, $\underline{u}_{\perp 2}$), and two thermal speeds ($\underline{w}_{\perp }$, $\underline{w}_{\parallel }$). A $_{\parallel }$ subscript indicates a quantity parallel to $\mathbf{B}$, and a _⊥_ subscript denotes a quantity perpendicular to $\mathbf{B}$. The fitting was done by obtaining a best guess of the parameters from numerical moments of the distribution function, and then using a least-squares method to minimise the cost function
  2 $$ C = \sum (f_{\mathrm{fit}} - f_{\mathrm{data}} )^{2} , $$
  where $f_{\mathrm{data}}$ was the experimentally measured distribution function, and the sum was taken over all velocity space points in $f_{\mathrm{data}}$. Note that the fitting was *not* done in logarithmic space.^2^ This means that the fitting was relatively insensitive to the tails of the distribution function, which was required to avoid that the lower amplitude proton beam influenced the fit to the proton core (see Figures 1 and 2 for a visual demonstration of this).

If no magnetic field values were available for any individual distribution function, it was still possible to locate the peak of the distribution, but the rotational symmetry axis of the bi-Maxwellian could not be determined. In this case, the fitting still took place in the instrument (non-rotated) frame of reference, but only the velocity component values were kept and thermal speeds and number density were discarded.

If the magnetic field direction varies significantly during the time it takes to measure a distribution function, the distribution is ‘smeared’ in the perpendicular direction, causing an overestimate of the field perpendicular temperature and number density (Verscharen and Marsch, 2011). If any two of the magnetic field vector measurements taken during the 32 seconds that it took the plasma instrument to measure a full distribution were more than 90 degrees apart, the number density and temperatures were considered unreliable and were not retained.

Figures 1 and 2 show examples of 2D cuts of the original distribution functions along with bi-Maxwellian fits in both the fast and slow solar wind. Out of a total of 2 216 195 original distribution functions, 1 869 275 were successfully fit with magnetic field values (providing density, velocity, and temperatures), and a further 227 436 were fitted without magnetic field values (providing only velocity).

## Comparison Between Moment and Corefit Datasets

**Figure 3:**
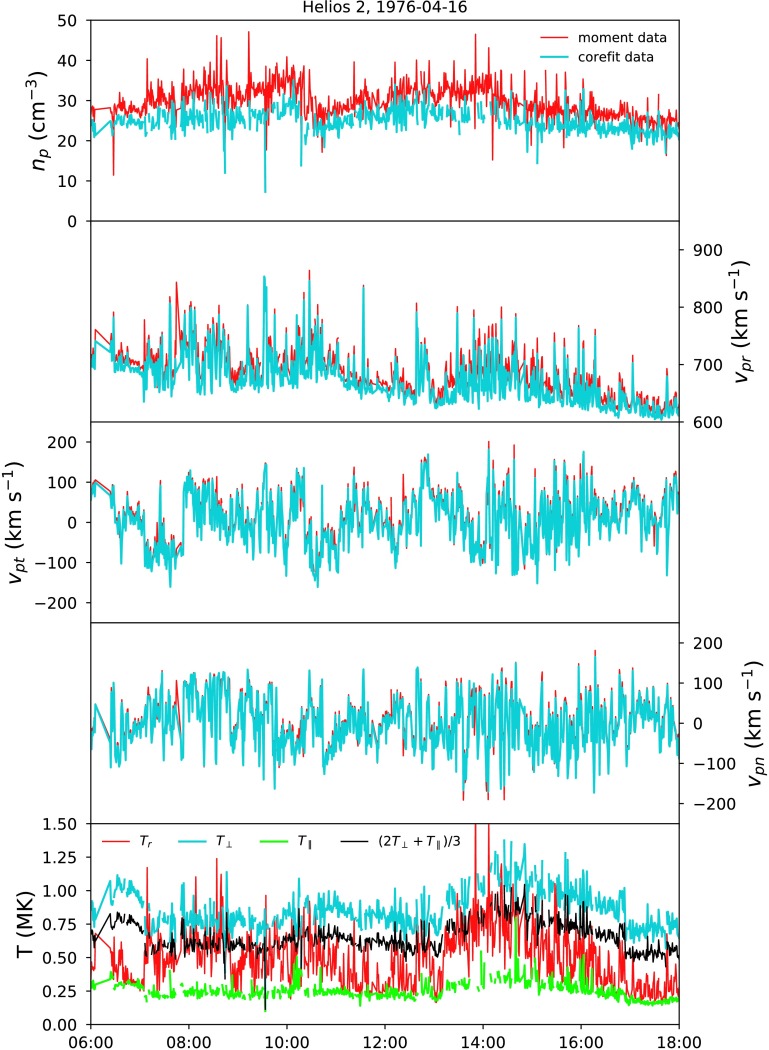
A 12-hour time series comparing the existing and new data. moment data are plotted in *red*, and corefit data in *blue* and *green*. From top to bottom, we show the proton number density, velocity components in an RTN coordinate system, and temperatures.

Figure 3 shows half a day of data comparing the already available moment dataset and the new corefit dataset described in Section 3. The main differences between the parameters in each dataset are discussed in the following sections.

**Figure 4:**
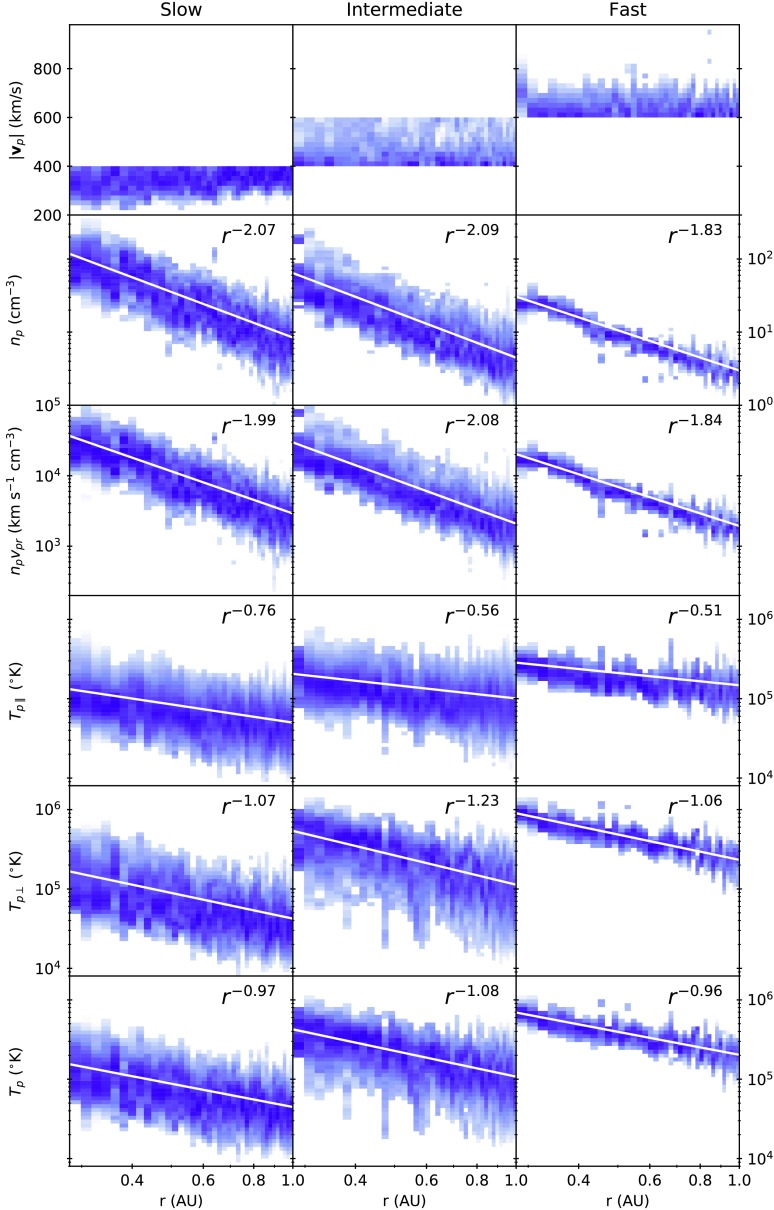
Radial trends of the proton core population. Histogram bins with counts greater than 100 were retained, and then normalised such that the bin values in each column sum to 1. *White lines* are power-law fits of Equation 5. Values of $A$ and $\gamma $ for each parameter are listed in Table 1.

### Number Density

The number density in the moment dataset contains contributions from the proton beam, so that is systematically higher than the corefit number density. The difference is typically around 20%, but can be as high as 50% at times. A time-series comparison is shown in the top panel of Figure 3.

### Velocity

The moment radial component of velocity is typically 1% larger than the corefit radial velocity component, due to the presence of the proton beam. The tangential and normal components are not affected by this, and the two datasets contain very similar values. A time-series comparison is shown in panels 2 – 4 of Figure 3.

### Temperature

The moment dataset contains only one proton temperature value. This value was calculated from the reduced 1D distribution function (the 3D distribution function integrated over all solid angles), and is the projection of the numerical temperature tensor along the radial direction, which means that it contains variable contributions from the true parallel and perpendicular temperatures of the protons depending on the local orientation of the magnetic field to the radial direction. The perpendicular and parallel temperatures in the corefit are not a function the magnetic field direction, and therefore provide a more meaningful characterisation of the true distribution function. A time-series comparison is shown in the bottom panel of Figure 3. The corefit total temperature, which can be calculated from the parallel and perpendicular temperatures via $T = ( 2T_{\perp } + T_{\parallel } ) / 3 $, is therefore a much more accurate characterisation of the average temperature than the moment dataset (see Figure 3, bottom panel). The moment temperature is typically 5%, which is higher than the corefit total temperature, but the difference is highly variable and ranges from 100% higher to 50% lower.

## New Radial Trends

In order to present the radial variation of parameters, the data were split into slow ($| \mathbf{v}_{\mathrm{p}} | < 400$ km s^−1^), intermediate ($400~\mbox{km}\,\mbox{s}^{-1} < | \mathbf{v}_{\mathrm{p}} | < 600~\mbox{km}\,\mbox{s}^{-1}$), and fast solar wind ($| \mathbf{v}_{\mathrm{p}} | > 600~\mbox{km}\,\mbox{s}^{-1}$). The radial dependence of each variable was parameterised by fitting a power law of the form

5 $$ f (r ) = A \biggl( \frac{r}{r_{0}} \biggr)^{-\gamma } $$

to the data between 0.29 and 1 AU, with $r_{0}$ = 1 AU, and $A$ and $\gamma $ as the two fit parameters. 2D histograms of the variables as a function of radial distance along with the fits are shown in Figure 4. The fitted values of $A$ and $\gamma $ for each variable and category of solar wind are reported in Table 1.

**Table 1:** Results of power-law fits as a function of radial distance. Fits are parameterised by Equation 5. $A$ is the 1 AU intercept, and $\gamma $ is the power-law exponent. See Figure 4 for a visual comparison of the fitted curves and underlying data.

	*γ*			*A*		
	Slow	Intermediate	Fast	Slow	Intermediate	Fast
$n_{\mathrm{p}}$	2.07	2.09	1.83	8.44 cm^−3^	4.47 cm^−3^	2.98 cm^−3^
$n_{\mathrm{p}} v_{\mathrm{pr}}$	1.99	2.08	1.84	2910 cm^−2^ s^−1^	2092 cm^−2^ s^−1^	1936 cm^−2^ s^−1^
$T_{\mathrm{p}\parallel }$	0.76	0.56	0.51	0.0500 MK	0.101 MK	0.148 MK
$T_{\mathrm{p}\perp }$	1.07	1.23	1.06	0.0423 MK	0.113 MK	0.233 MK
$T_{\mathrm{p}}$	0.97	1.08	0.96	0.0447 MK	0.108 MK	0.203 MK

From the new radial trends the following well-known results are reproduced:

- The number density in the slow solar wind is larger and more variable than in the fast solar wind.
- The number density decreases faster than a simple $1/r^{2}$ constant speed decrease in the slow solar wind ($\gamma = 2.07$) and slower than simple radial expansion in the fast solar wind ($\gamma =1.83$). This is most likely due to the slow solar wind accelerating and the fast solar wind decelerating between 0.3 AU and 1 AU.
- The radial flux almost follows a $1/r^{2}$ decrease in the slow solar wind ($\gamma = 1.99$), but decreases more slowly in the fast solar wind ($\gamma = 1.84$).
- The slowest solar wind at 0.3 AU ($\sim 200$ km s^−1^) is accelerated up to a higher minimum ($\sim 250$ km s^−1^) by 1 AU.

In addition, the trends successfully reproduce a number of features in the radial evolution of temperatures that were initially observed by Marsch *et al.* (1982b):

- Both $T_{\mathrm{p}\perp }$ and $T_{\mathrm{p}\parallel }$ are higher and less variable in the fast solar wind.
- $T_{\mathrm{p}\perp }$ decreases faster with radial distance than $T_{\mathrm{p}\parallel }$.
- $T_{\mathrm{p}\perp }$ decreases faster with radial distance in the fast solar wind (than in the slow wind), whereas $T_{\mathrm{p}\parallel }$ decreases faster with radial distance in the slow solar wind (than in the fast wind).
- $T_{\mathrm{p}\perp }$ and $T_{\mathrm{p}\parallel }$ both decrease more slowly than a single adiabatic prediction ($\gamma = 5/3$).
- $T_{\mathrm{p}\perp }$ decreases more slowly than the Chew, Goldberger, and Low (1956) double adiabatic prediction in a radial magnetic field ($\gamma = 2$), but $T_{\mathrm{p}\parallel }$ decreases faster than the prediction ($\gamma = 0$).

Marsch *et al.* (1982b) and Hellinger *et al.* (2011, 2013) have previously performed similar analyses of the *Helios* data to extract the parallel and perpendicular temperatures, using numerical moments of the distribution function instead of analytical fits. This means that they did not separate out the contributions from the proton core and beam. The presence of a beam led to higher $T_{\mathrm{p}\parallel }$ values in both datasets than in ours. Matteini *et al.* (2013) have also carried out a similar bi-Maxwellian analysis of the proton core using *Ulysses* data measured at 1.5 – 5.4 AU (Neugebauer *et al.*, 2001). Because *Ulysses* sampled high heliographic latitudes, it primarily observed the radial evolution of the fast solar wind. $T_{\perp }$ decreases as $r^{-0.9}$ at large distances, close to the *Helios* evolution of $r^{-1}$, whereas $T_{\parallel }$ decreases much more slowly ($r^{-0.2}$) when compared to *Helios* ($r^{-0.5}$). This is probably due to the increasing influence of non-adiabatic processes such as temperature anisotropy and drift instabilities at large heliocentric distances (Matteini *et al.*, 2013).

Finally, we note that combining data from a wide range of times and locations into single radial fits means that the data do not sample how a single parcel of plasma evolves as it propagates radially outwards. Nonetheless, this type of analysis is useful for indicating the average behaviour of the solar wind.

## Conclusions

We have presented the method and results of a complete reprocessing of the original *Helios* solar wind ion distribution functions, measured between 0.29 and 1 AU. The resulting dataset has been made freely available on the *Helios* data archive (http://helios-data.ssl.berkeley.edu/) for other researchers to use, and the code we used to fit the distributions functions has also been made available, making the dataset reproducible. The new data provide a benchmark of how the proton core evolves in the inner heliosphere. This dataset forms an important resource against which *in-situ* data from the upcoming *Parker Solar Probe* and *Solar Orbiter* missions will be compared to study variations in the solar wind on decadal timescales.

## Footnotes

1 Available at ftp://cdaweb.gsfc.nasa.gov/pub/data/helios/helios1/merged/ and ftp://cdaweb.gsfc.nasa.gov/pub/data/helios/helios2/merged/.

2 *I.e.*, minimising $\sum \log |f_{\mathrm{fit}} - f_{\mathrm{data}} |$.
